# Inhibition of Bromodomain and Extraterminal Domain (BET) Proteins by JQ1 Unravels a Novel Epigenetic Modulation to Control Lipid Homeostasis

**DOI:** 10.3390/ijms21041297

**Published:** 2020-02-14

**Authors:** Claudia Tonini, Mayra Colardo, Barbara Colella, Sabrina Di Bartolomeo, Francesco Berardinelli, Giuseppina Caretti, Valentina Pallottini, Marco Segatto

**Affiliations:** 1 Department of Science, University of Rome “Roma Tre”, Viale Marconi 446, 00146 Rome, Italy; claudia.tonini@uniroma3.it (C.T.); francesco.berardinelli@uniroma3.it (F.B.); valentina.pallottini@uniroma3.it (V.P.); 2 Department of Bioscience and Territory, University of Molise, Contrada Fonte Lappone, 86090 Pesche (Is), Italy; m.colardo@studenti.unimol.it (M.C.); b.colella@studenti.unimol.it (B.C.); sabrina.dibartolomeo@unimol.it (S.D.B.); 3 Department of Biosciences, University of Milan, Via Celoria 26, 20133 Milan, Italy; giuseppina.caretti@unimi.it

**Keywords:** BET proteins, cell proliferation, cholesterol, epigenetics, HMGCR, JQ1, LDLr, lipid metabolism, SREBP, TMEM97

## Abstract

The homeostatic control of lipid metabolism is essential for many fundamental physiological processes. A deep understanding of its regulatory mechanisms is pivotal to unravel prospective physiopathological factors and to identify novel molecular targets that could be employed to design promising therapies in the management of lipid disorders. Here, we investigated the role of bromodomain and extraterminal domain (BET) proteins in the regulation of lipid metabolism. To reach this aim, we used a loss-of-function approach by treating HepG2 cells with JQ1, a powerful and selective BET inhibitor. The main results demonstrated that BET inhibition by JQ1 efficiently decreases intracellular lipid content, determining a significant modulation of proteins involved in lipid biosynthesis, uptake and intracellular trafficking. Importantly, the capability of BET inhibition to slow down cell proliferation is dependent on the modulation of cholesterol metabolism. Taken together, these data highlight a novel epigenetic mechanism involved in the regulation of lipid homeostasis.

## 1. Introduction

The homeostatic regulation of lipid metabolism is essential for the maintenance of key cellular processes involved in a plethora of biological functions. Fatty acids constitute the major energy source and are fundamental constituents of cell membranes [1]. In addition, they serve as substrates for cellular phospholipases, which convert these compounds into pivotal signalling factors implicated in anti- and pro-inflammatory actions [2]. Similarly, cholesterol exerts both structural and functional roles, being the precursor of steroid hormones, vitamin D and bile acids, and regulating the assembly of specialized membrane microdomains called lipid rafts and caveolae [3].

During last decades, it has become increasingly clear that lipid homeostasis is crucial for cell growth and proliferation [4,5], as membrane biosynthesis requires the coordinated assembly of different lipid species during cell division, involving the concerted regulation of lipid biosynthesis, uptake, subcellular localization and turnover [6]. For these reasons, the body employs an intricate homeostatic network to regulate the availability of lipids for cells and tissues. This network mainly operates in the liver, where the major part of lipid metabolism takes place [3]. Hepatic cells are the principal site for lipid biosynthesis, determined by the establishment of a complex series of enzymatic reactions; in particular, 3-hydroxy-3-methylglutaryl Coenzyme A reductase (HMGCR) and Acyl Coenzyme A carboxylase (ACC) represent the key and rate-limiting enzymes for cholesterol and fatty acid synthesis, respectively [7,8]. Considering their central role in lipid biosynthesis, both HMGCR and ACC are tightly regulated at both short- and long-term levels. Short-term regulation is controlled by phosphorylative events, which negatively affect the activation state of the enzymes [9]. Conversely, the long-term transcriptional regulation of the protein machinery involved in lipid metabolism is mediated by sterol regulatory element binding proteins (SREBPs). When intracellular sterol content is reduced, SREBPs precursors are proteolytically processed to form the NH(2)-terminal fragment that enters the nucleus (nuclear, nSREBP) and induces the transcription of genes coding for lipogenic enzymes, such as HMGCR and ACC [9,10]. Notably, SREBPs also promote the extracellular uptake of lipoprotein-derived lipids by eliciting the transcription of lipoprotein receptors, such as low density lipoprotein receptor (LDLr) and scavenger receptor class B type 1 (SR-B1) [11,12].

Lipid abnormalities play a crucial role in a plethora of pathological conditions, such as cardiovascular diseases, neurodevelopmental alterations, neurodegenerative and lipid storage disorders [13,14,15]. Despite the widespread use of lipid-lowering medications, effective pharmacological approaches are still lacking for several lipid metabolism-related disorders. Thus, there is still a need to better dissect the regulatory mechanisms of lipid homeostasis in order to identify novel molecular targets that could be useful for designing promising therapeutic treatments.

Bromodomain and extra-terminal domain (BET) proteins, comprising BRD2, BRD3 and BRD4, are epigenetic readers recruited to the chromatin by the presence of acetylated histones, thereby regulating gene expression [16]. The involvement of these epigenetic sensors has been extensively characterized in cancer and inflammation, because of their incontrovertible role in the transcriptional modulation of oncogenes and mediators of the immune response [17,18]. BET proteins attracted considerable interest in biomedical research, as they are extremely druggable by potent and specific inhibitors. In this context, BET inhibition exerts anti-proliferative activity in different cancer cells and shows outstanding anti-inflammatory properties in a number of physiopathological conditions [17,19,20]. Recently, experimental evidence also demonstrated that the activity of BET proteins may be extended to the regulation of metabolic processes. For instance, BET inhibition strongly affects protein homeostasis, autophagy induction, reactive oxygen species (ROS) and glucose metabolism [17,21,22,23,24]. Conversely, the prospective role exerted by BET proteins on lipid metabolism is still poorly characterized. Microarray analysis suggested that the BET inhibitor RVX-208 induces changes in ApoA1 and high density lipoprotein (HDL) levels [25]. Coherently, overexpression of BET proteins increased cholesterol biosynthesis [26]. Furthermore, it has been observed that BET inhibition efficiently counteracted plasma low density lipoprotein (LDL) alterations in a mouse model of cancer cachexia [17]. Despite this evidence, no studies systematically addressed the involvement of BET proteins in the modulation of the main proteins and enzymes controlling the regulatory machinery of lipid metabolism. A better comprehension of these mechanisms is essential to identify novel physiological regulatory pathways and to select innovative therapeutic targets. Here, we provide a proof of concept study, aimed at evaluating the putative role of BET modulation on lipid homeostasis. To reach this objective, we took advantage of a loss-of-function approach by using JQ1 as a potent and specific inhibitor of BET protein activity [27]. The effects induced by BET inhibition were mainly evaluated in HepG2 cell line, a human liver-derived cell culture model widely used to assess lipid homeostasis [28,29,30].

## 2. Results

### 2.1. BET Inhibition Decreases Lipid Content in HepG2 Cells

**Figure 1:**
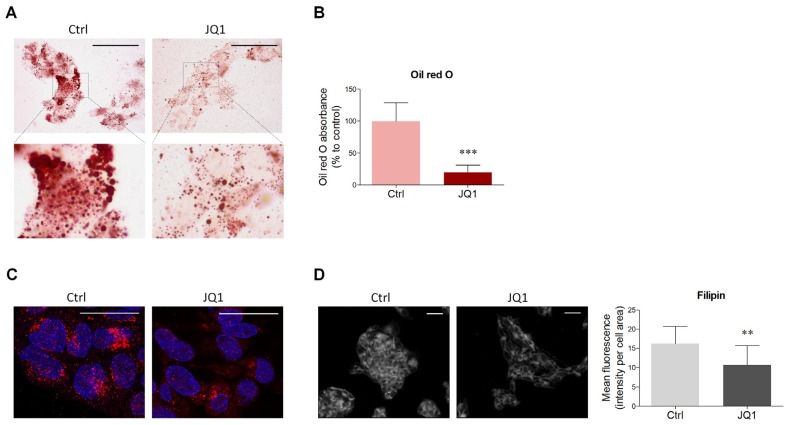
Effects of bromodomain and extraterminal domain (BET) inhibition by JQ1 on intracellular lipids in HepG2 cells. (**A**) HepG2 cells were treated with vehicle (Ctrl) or JQ1 (0.4 µM), and after 48 hours they were stained with Oil Red O as described in the Materials and Methods Section to visualize the intracellular content of neutral lipids. *n* = 6 different experiments. Scale bar: 50 µm (**B**) HepG2 cells were treated as in (A), and Oil Red O was extracted with isopropanol. The eluted dye was then quantified by spectrophotometry to evaluate the amount of neutral lipids. *n* = 6 different experiments. (**C**) Vehicle- and JQ1-treated HepG2 cells were fixed and stained with antibody against Plin2 (red). DAPI was used as a nuclear counterstain. Scale bar: 25 µm (**D**) Representative image (left panel) and quantification of the mean fluorescence intensity (right panel) of filipin staining performed on HepG2 cells treated with vehicle and JQ1 for 48 hours. *n* = 5 different experiments. Scale bar: 50 µm. Data represent means ± SD. Statistical analysis was performed by using unpaired Student’s t test. ** *p* < 0.01; *** *p* < 0.001.

In order to evaluate the putative involvement of BET inhibition on lipid homeostasis, the effect of JQ1 on cellular lipid content was firstly assessed. HepG2 cells were treated with JQ1 for 48 hours, and Oil Red O staining was employed as a widely used and accurate method to measure neutral lipids [31]. Descriptive evaluation highlighted that the number of lipid droplets, as well as their size, appeared reduced in JQ1-treated cells (Figure 1A). This result corroborated the quantitative assessment of lipid content estimated by Oil Red O absorbance, which showed a significant five-fold decrease upon BET inhibition (Figure 1B). Coherently, immunofluorescence intensity of the lipid droplet marker perilipin-2 (Plin2) was found to be lower 48 hours after pharmacological BET blockade (Figure 1C). Notably, JQ1 was also effective in reducing the amount of intracellular cholesterol, as observable by filipin staining and quantification (Figure 1D). Thus, BET inhibition significantly lowers lipid content in HepG2 cells.

### 2.2. BET Inhibition by JQ1 Modulates the Expression of Proteins and Enzymes Involved in Lipid Metabolism

**Figure 2:**
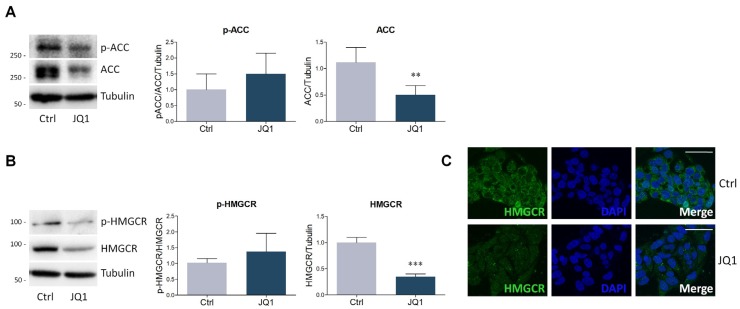
Evaluation of BET inhibition on lipid biosynthesis enzymes. (**A**) Representative Western blot (left panel) and densitometric analysis of phosphorylated Acyl Coenzyme A carboxylase (ACC) (P-ACC, ser79) and total ACC in HepG2 cells treated with vehicle (Ctrl) or JQ1 (0.4 µM) for 48 hours. *n* = 6 independent experiments. Tubulin was employed as a housekeeping protein to normalize protein loading. (**B**) Representative Western blot (left panel) and densitometric analysis (right panel) of phosphorylated 3-hydroxy-3-methylglutaryl Coenzyme A reductase (HMGCR) (p-HMGCR, ser872) and total HMGCR in HepG2 cells treated with vehicle (Ctrl) or JQ1 (0.4 µM) for 48 hours. *n* = 7 independent experiments. Tubulin served as a housekeeping protein to normalize protein loading. (**C**) Immunofluorescence staining of HMGCR (green) of HepG2 cells treated as in (B). Nuclei were counterstained with DAPI. *n* = 3 different experiments. Scale bar: 50 µm. Data are expressed as means ± SD. Statistical analysis was carried out by using unpaired Student’s t test. ** *p* < 0.01; *** *p* < 0.001.

To understand the cellular mechanisms underlying the reduction of lipid content induced by BET inhibition, the prospective modulation of proteins belonging to the lipid metabolism machinery were assessed. The analysis was initially focused on ACC and HMGCR, the rate-limiting enzymes involved in fatty acid and cholesterol biosynthesis, respectively. Western blot analysis revealed that JQ1 treatment significantly decreased ACC protein expression if compared to vehicle-treated HepG2 cells (Figure 2A). However, no changes were observed in the ratio between the phosphorylated fraction of ACC and its total levels, suggesting that BET inhibition modulated the protein amount of the enzyme without influencing its activation state by inhibitory phosphorylation. Similar results were obtained by analyzing HMGCR; in fact, JQ1 administration strongly reduced the protein levels of the enzyme without affecting its phosphorylation state (Figure 2B). The effect of BET inhibition on HMGCR expression was further confirmed by confocal analysis, showing an overall decrease of immunofluorescence intensity in JQ1-treated HepG2 with respect to control cells (Figure 2C).

**Figure 3:**
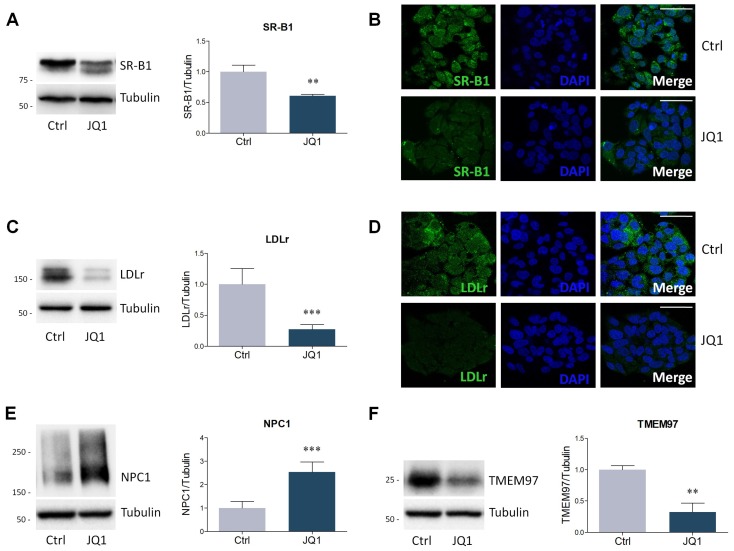
Expression of proteins involved in extracellular lipid uptake and intracellular cholesterol trafficking following JQ1 (0.4 µM) administration to HepG2 cells for 48 hours. (**A**) Representative Western blot (left panel) and densitometric analysis (right panel) of SR-B1. *n* = 6 independent experiments. (**B**) Immunofluorescence analysis of SR-B1 (green). Nuclei were counterstained with DAPI. *n* = 3 different experiments. Scale bar: 50 µm. (**C**) Representative Western blot (left panel) and densitometric analysis (right panel) of LDLr. *n* = 5 independent experiments. (**D**) LDLr immunofluorescence (green). Nuclei were counterstained with DAPI. *n* = 3 different experiments. (**E–F**) Representative Western blots and densitometric analysis of NPC1 and TMEM97. Tubulin was chosen as loading control. *n* = 6 independent experiments. Data represent means ± SD. Statistical analysis was performed by using unpaired Student’s t test. ** *p* < 0.01; *** *p* < 0.001.

Lipid homeostasis is guaranteed by a delicate equilibrium between biosynthesis and extracellular uptake. The latter process is mainly operated by LDLr, which internalizes LDL through receptor-mediated endocytosis [32]. In addition to LDLr, hepatic cells also express SR-B1, a multiligand receptor that binds several lipoproteins, including HDL and LDL [33]. Considering their pivotal role in the physiological regulation of lipid metabolism, the prospective effects mediated by BET inhibition were also assessed for these two lipoprotein receptors. SR-B1 expression was significantly repressed by JQ1 treatment, as observed by Western blot and immunofluorescence data (Figure 3A,B). Similarly, BET inhibition determined a three-fold reduction in LDLr expression levels (Figure 3C). Immunofluorescence microscopy confirmed this result, being LDLr barely detectable in JQ1-treated HepG2 cells when compared to vehicle-treated cells (Figure 3D).

Upon binding to lipoprotein receptors, LDL are internalized and transported throughout the endocytic pathway to lysosomes, where cholesteryl esters can be hydrolyzed by acid lipases [34]. Subsequently, unesterified cholesterol exits the lysosomal compartment, through the activity of NPC1, and is delivered to the plasma membrane and the endoplasmic reticulum (ER) [35]. Because of its essential role in intracellular cholesterol trafficking, Niemann-Pick type C1 (NPC1) protein levels were assessed in this study. BET inhibition by JQ1 strongly enhances NPC1 protein expression in HepG2 cells (Figure 3E). Interestingly, the rise in NPC1 levels was accompanied by a significant reduction of transmembrane protein 97 (TMEM97) protein content, also known as the sigma-2 receptor (Figure 3F), which has already been involved in NPC1 regulation [36].

**Figure 4:**
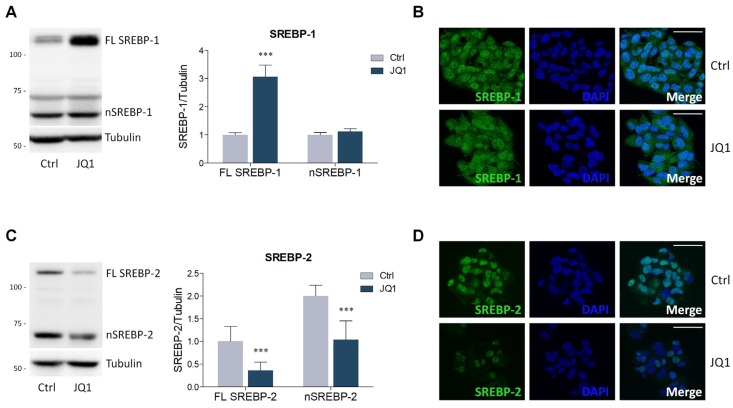
BET inhibition alters sterol regulatory element binding proteins (SREBPs) expression in HepG2 cells treated with JQ1 (0.4 µM) for 48 hours. (**A**) Representative Western blot (left panel) and densitometric analysis (right panel) of SREBP-1. FL SREBP-1 (Full-length SREBP-1); nSREBP-1 (nuclear SREBP-1). *n* = 6 independent experiments. Tubulin was employed for control loading. (**B**) SREBP-1 immunofluorescence staining (green) in HepG2 cells. Nuclei were counterstained with DAPI. *n* = 3 different experiments. Scale bar: 50 µm. (**C**) Representative Western blot (left panel) and densitometric analysis (right panel) of SREBP-2. FL SREBP-2 (Full-length SREBP-2); nSREBP-2 (nuclear SREBP-2). *n* = 6 independent experiments. Tubulin was used as a housekeeping protein. (**D**) Immunofluorescence analysis of SREBP-2 (green). Nuclei were counterstained with DAPI. *n* = 3 different experiments. Scale bar: 50 µm. Data represent means ± SD. Statistical analysis was assessed by using unpaired Student’s t test. *** *p* < 0.001.

Most proteins involved in lipid metabolism are under the transcriptional control of SREBPs. Considering the effects of BET inhibition evaluated in this work, the expression of SREBP-1 and SREBP-2 was estimated. JQ1 treatment induced a significant increase of SREBP-1 precursor (full-length, FL SREBP-1). However, this effect was not paralleled by a concurrent modification in the nuclear and transcriptionally active fragment of SREBP-1 (nSREBP-1), as its expression is similar between the two experimental groups (Figure 4A). Morphological analysis corroborated this evidence, revealing a slightly higher fluorescence intensity in the cytosolic compartment in JQ1-treated cells, consistent with the increase of FL SREBP-1 observed by the Western blot. On the contrary, no differences were detected in the number and intensity of the stained nuclei, reflecting the lack of significant alterations in the amount of nSREBP-1 (Figure 4B). Differently from SREBP-1, BET inhibition reduced the expression of both precursor (FL SREBP-2) and nuclear SREBP-2 (nSREBP-2) (Figure 4C). SREBP-2 immunofluorescence revealed a predominant nuclear staining, and a weak signal in the cytoplasmic compartment. Notably, JQ1 administration markedly decreased the intensity and the number of nuclei stained for SREBP-2 (Figure 4D).

**Figure 5:**
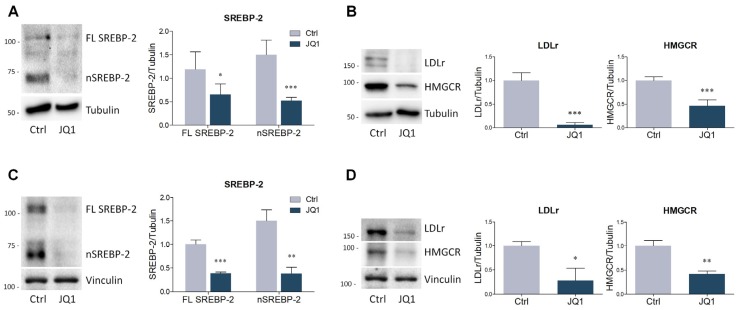
BET inhibition modulates the expression of SREBP-2, HMGCR and LDLr in different cell lines. (**A–B**) Representative Western blot and densitometric analysis of SREBP-2, HMGCR and LDLr in differentiated N1E-115 treated with JQ1 (0.1 µM) for 48 hours. (**C–D**) Representative Western blot and densitometric analysis of SREBP-2, HMGCR and LDLr in primary human fibroblasts treated with JQ1 (0.4 µM) for 48 hours. *n* = 3 different experiments. Tubulin and vinculin were used as loading control. Data represent means ± SD. Statistical analysis was assessed by using unpaired Student’s t test. * *p* < 0.05, ** *p* < 0.01, *** *p* < 0.001.

To further strengthen the effects induced by JQ1 on lipid homeostasis in HepG2 cells, we recapitulated the most relevant findings in different cell lines. As expected, BET blockade significantly suppressed the expression of SREBP-2 and of its targets LDLr and HMGCR also in the neuroblastoma cell line N1E-115 (Figure 5A,B) and in primary culture of human fibroblasts (Figure 5C,D).

Overall, these data indicate that BET inhibition deeply affects the main proteins involved in lipid biosynthesis, uptake and intracellular transport.

### 2.3. BET Inhibition Affects Cell Proliferation in a Cholesterol-Dependent Manner

**Figure 6:**
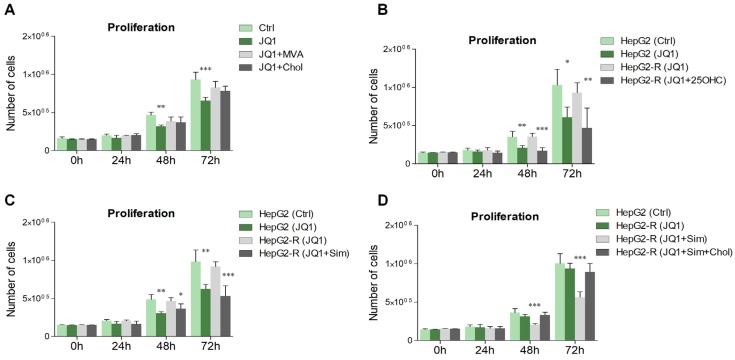
BET inhibition influences cell proliferation through cholesterol metabolism. (**A**) HepG2 cells were seeded in 6-well plates (150,000 cells for each well) and were treated with JQ1 (0.4 µM), mevalonate (MVA, 100 µM) and cholesterol (chol, 50 µM) for 72 hours. Cell counts were conducted over time with a hemocytometer. (**B–C**) Cell proliferation was evaluated in JQ1-sensitive (HepG2) and JQ1-resistant (HepG2-R) cells. JQ1-resistant and -sensitive HepG2 cells were treated with the BET inhibitor for 72 hours, and additional groups of HepG2-R cells were co-stimulated with JQ1+25-hydroxycholesterol (25OHC, 20 µM) or JQ1+simvastatin (Sim, 1 µM). Other cells were treated with vehicle and served as control (Ctrl). (**D**) Cell proliferation was evaluated in JQ1-sensitive (HepG2) and JQ1-resistant (HepG2-R) cells. JQ1-resistant cells were constantly stimulated with the BET inhibitor. Additional groups of HepG2-R cells were co-stimulated with JQ1+simvastatin (Sim, 1 µM) or JQ1+Sim+cholesterol (Chol, 50 µM). *n* = 4 independent experiments. Data represent means ± SD. Statistical analysis was assessed by using one-way ANOVA, followed by Dunnett’s post hoc. * *p* < 0.05; ** *p* < 0.01; *** *p* < 0.001.

The maintenance of a proper amount of lipids, and in particular of cholesterol, is crucial for several biological processes, such as cell growth and cell proliferation [4,37]. In addition, it has been extensively demonstrated that BET inhibition promotes the suppression of cell proliferation in a number of normal and cancer cell types [19,38,39]. Results collected in this work are in agreement with previous reports, demonstrating that JQ1 treatment significantly slowed down the proliferation rate of HepG2 cells starting at 48 hours from the pharmacological treatment (Figure 6A). Remarkably, the cell growth rate was rescued when JQ1 was co-administered with mevalonate (MVA), the product of the reaction catalyzed by HMGCR. The involvement of cholesterol biosynthesis in the anti-proliferative effects mediated by BET inhibition was further supported by the co-administration of cholesterol to JQ1-treated cells that, similarly to MVA addition, was able to restore cell proliferation. In order to delve deeper into the effects exerted by BET inhibition on cell proliferation and cholesterol modulation, we generated a HepG2 lineage with acquired resistance to JQ1 (HepG2-R) by continuously treating cells with increasing doses of the drug. While JQ1 decreased the number of JQ1-sensitive cells as previously shown, no effects were induced in HepG2-R upon drug administration (Figure 6B). Importantly, the restoration of cell proliferation observed in HepG2-R cells was completely abolished by 25-hydroxycholesterol (25OHC) treatment, a well-known methodological approach to mediate HMGCR degradation through the activation of a feedback mechanism [40]. Similar results were also obtained by blocking HMGCR activity with simvastatin, a potent inhibitor of cholesterol biosynthesis (Figure 6C). Because statins suppress the production of isoprenoid intermediates in the cholesterol biosynthetic pathway, they can exert a plethora of pleiotropic actions independently from cholesterol decrease [8]. To confirm a direct involvement, cholesterol was then administered to HepG2-R cells co-treated with JQ1 and simvastatin. Cholesterol supplementation efficiently prevented the reduction of cell proliferation induced by simvastatin in HepG2-R cells, thus restoring the acquired resistance to JQ1 as a function of cell growth (Figure 6D).

**Figure 7:**
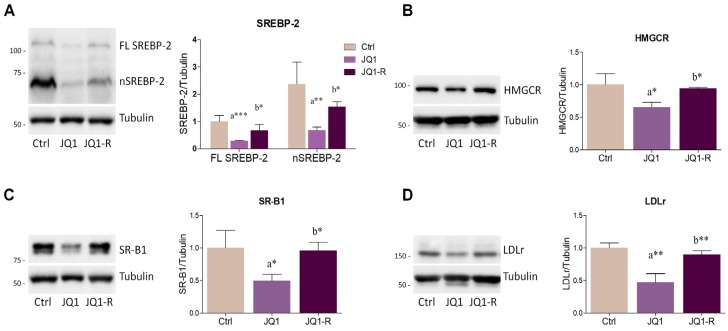
JQ1 resistance is accompanied by the upregulation of proteins controlling cholesterol metabolism. (**A–D**) Representative Western blots and densitometric analysis of SREBP-2, HMGCR, SR-B1and LDLr in JQ1-sensitive cells treated with vehicle (Ctrl) or JQ1 (0.4 µM), and in HepG2-resistant (JQ1-R) cells constantly stimulated with JQ1 (0.4 µM). Experiments were performed after 48 hours. *n* = 4 independent experiments. Tubulin served as loading control. Data represent means ± SD. Statistical analysis was assessed by using one-way ANOVA followed by Tukey’s post-hoc. * *p* < 0.05; ** *p* < 0.01; *** *p* < 0.001. “a” indicates statistical significance versus control group (Ctrl); “b” indicates statistical significance compared to JQ1 group.

Overall, these results suggested that the anti-proliferative effects elicited by JQ1 can be mediated by the suppression of cholesterol metabolism and that the acquisition of resistance to BET inhibition may be accompanied by adaptive changes in the main proteins controlling cholesterol homeostasis. Western blot data confirmed this hypothesis, highlighting that both FL SREBP-2 and nSREBP-2 levels were increased in JQ1-resistant HepG2 cells (Figure 7A). Coherently, the expression of SREBP-2 target genes HMGCR, SR-B1 and LDLr was properly restored at the level of control cells in HepG2-R cells (Figure 7B–D). Taken together, these results suggest that the impact of HepG2 proliferation mediated by BET inhibition is dependent on cholesterol metabolism.

## 3. Discussion

The homeostatic maintenance of lipid metabolism plays a fundamental role in assuring the correct development of cellular functions. As a consequence, alterations in the mechanisms controlling lipid balance may be associated to several pathological conditions, ranging from cardiovascular diseases and lipidosis to neurodegenerative and neurodevelopmental diseases [13,14,15,41]. The discovery of effective lipid-lowering medications, such as statins and fibrates, have revolutionized the treatment and the clinical outcomes of different lipid disorders [42]. Unfortunately, numerous diseases associated to lipid disturbances do not yet have a resolutive therapy. The obstacles in designing efficient pharmacological approaches relies on the fact that cellular and molecular mechanisms regulating lipid metabolism are not completely elucidated.

In this context, it is important to delve deeper into the regulatory mechanisms of lipid homeostasis, with the attempt to unravel potential etiopathological factors and novel molecular targets that could be useful to set up promising therapies. In the last few years, several reports highlighted that epigenetic factors may play crucial roles in the control of lipid homeostasis. For instance, it is becoming increasingly clear that histone deacetylases (HDAC) and microRNAs exert a crucial modulatory activity in lipid and energy metabolism [43,44,45,46,47]. On the contrary, the involvement of BET proteins in the regulation of lipid homeostasis is still elusive. Thus, in this work, we evaluated the effects of BET inhibition in the expression of the main proteins controlling lipid metabolism.

Collectively, our results demonstrated that BET inhibition induced an overall suppression of lipid metabolism. In particular, JQ1 administration decreased the intracellular lipid content and reduced the expression of biosynthetic enzymes (ACC and HMGCR) as well as of receptors involved in lipoprotein uptake (LDLr and SR-B1). BET inhibition also induced a strong downregulation in the expression of TMEM97. This conserved integral membrane protein has been identified as an important modulator of cholesterol levels and, similarly to other proteins coordinating lipid metabolism, is a SREBP-2 target gene [36,48,49]. It is well described that SREBPs isoforms possess different roles in lipid biosynthesis. SREBP-1 isoforms are mostly devoted to the regulation of fatty acid and triglyceride metabolism, whereas SREBP-2 is relatively selective in activating cholesterol-related genes [50,51]. Coherently with these notions, the JQ1-mediated suppression of HMGCR, LDLr, SR-B1 and TMEM97 was paralleled by a concurrent decrease of both the full-length and the nuclear active fraction of SREBP-2. Interestingly, BET inhibition was also responsible for a significant decrease of ACC expression, which, however, was not adequately accompanied by changes in nSREBP-1 levels. Indeed, the levels of SREBP-1 precursor were significantly augmented, probably representing a transcriptional attempt to counteract the marked decrease of the SREBP-2 isoform. Conversely, the expression of the nuclear and transcriptionally active fragment of SREBP-1 was unaltered upon JQ1 treatment, excluding its involvement in the modulation of ACC levels. Even though ACC transcription is preferentially controlled by SREBP-1 isoforms [50], it has been observed that SREBP-2 can equally influence ACC transcription [52], supporting the notion that the reduction of ACC levels observed in this work may be ascribable to the suppression of SREBP-2 mediated by JQ1. In addition, BET blockade led to a rise in NPC-1 protein levels. Literature data illustrated that a reduction of TMEM97 increases NPC-1 protein expression by a post-translational mechanism [36], and suggest that the suppression of TMEM97 could explain the build-up of NPC-1 observed in this study following JQ1 administration. Consistent with JQ1-mediated decrease of LDLr expression, NPC1 induction may also represent a refined compensatory response to contrast the prospective decrease of LDL-cholesterol uptake. Furthermore, it cannot be excluded that BET inhibition directly affects the transcription of *NPC1*, as well as of other genes involved in the maintenance of lipid homeostasis.

Subsequently, the prospective contribution of lipid metabolism in the anti-proliferative effects induced by JQ1 administration was evaluated. It has been extensively reported that BET inhibition exerts a remarkable reduction in cell proliferation by hindering the transcription of oncogenes such as c-Myc [53], and by suppressing the activation of pro-survival and growth signalling kinases like Akt [26]. In this study, we highlight for the first time a novel mechanism by which BET inhibition modulates cell proliferation in a cholesterol-dependent manner. Indeed, the administration of both MVA and cholesterol to culture medium efficiently abolished the delay in cell proliferation induced by JQ1. Consistently, the generation of HepG2-R cells further corroborated this evidence, as the acquisition of resistance to JQ1 is associated to the compensatory increase in the expression of proteins belonging to cholesterol metabolism, and to the restoration of cell proliferation rate.

Overall, these data suggest that BET inhibition reduces lipid content by hampering SREBP-2 expression and processing, which result in a reduced expression of target genes involved in lipid biosynthesis, uptake and intracellular trafficking.

Despite the fact that more efforts should be done in order to better clarify the specific contribution of each BET protein in the regulation of lipid metabolism, this work provides the proof of principle that epigenetic pathways, influenced by BET protein activity, represent novel physiological mechanisms to control lipid homeostasis. These findings may also set the basis for designing innovative therapeutic approaches aimed at ameliorating the functional outcomes of several disorders characterized by lipid unbalances.

## 4. Materials and Methods 

### 4.1. Cell Cultures and Generation of HepG2 Resistant to JQ1

N1E-115 neuroblastoma cells were cultured at 5% CO2 in DMEM medium at high glucose, containing 10% (*v/v*) foetal calf serum, L -glutamine (2 mM), and added with penicillin/streptomycin solution. Cells were then seeded at 50% confluency and were induced to differentiate for four hours by adding 2% dimethyl sulfoxide. Differentiated N1E-115 cells were treated with JQ1 (0.1 µM) or vehicle (DMSO, dilution 1:1000 in cell culture media) for 48 hours.

Human foetal foreskin fibroblasts (HFFF2) were grown at 5% CO2 in DMEM (high glucose) additioned with 10% foetal calf serum, L -glutamine (2 mM), and penicillin/streptomycin solution. HFFF2 (60% confluency) were then treated with 0.4 µM JQ1 for 48 hours. Cells treated with vehicle (DMSO, dilution 1:1000 in cell culture media) served as control.

HepG2 cells were routinely cultured at 5% CO2 in DMEM medium at high glucose, containing 10% (*v/v*) foetal calf serum, L -glutamine (2 mM), and added with penicillin/streptomycin solution. Cells were passaged every 3 days and medium changed every 2–3 days. All the experiments were performed with cell confluency of 60%–70%. DMSO stock of 4 mM JQ1 was diluted 1:1000 in cell culture media to obtain the final concentration of 0.4 µM JQ1. Experiments were then performed 48 hours after JQ1 stimulation. Cells treated with vehicle (DMSO, dilution 1:1000 in cell culture media) served as control. The generation of HepG2 cells resistant to JQ1 was carried out by slightly modifying the protocol provided by Gobbi and colleagues [54]. HepG2 resistant cells were selected by continuously applying increasing doses of JQ1, starting from 50 nM and enhancing the drug concentration every 1–2 weeks for 2 months of total treatment. Surviving JQ1-resistant cells were then maintained at 0.4 µM JQ1 throughout. For cell proliferation experiments, chemicals were purchased from Sigma-Aldrich and used at the following concentrations: JQ1 (0.4 µM), mevalonate (100 µM), cholesterol (50 µM), simvastatin (1 µM), 25-hydroxycholesterol (20 µM).

### 4.2. Oil Red O Staining and Quantification

For the evaluation of lipid content, HepG2 cells were seeded in 12-well plates. Briefly, after 48 hours of JQ1 treatment, cells were fixed in paraformaldehyde (4% solution) for 10 minutes and gently rinsed twice with PBS. Sixty per cent isopropanol was added to fixed cells for 5 minutes, and then washed 2 times with distilled water. Cells were stained with 1 mL of the Oil Red O solution (Sigma-Aldrich, Milan, Italy) for 15 minutes at room temperature with continuous gentle shaking. Subsequently, wells were then rinsed 3 times with distilled water, until no excess stain was seen. Stained cells were visualized in brightfield using an Olympus BX 51 microscope (Olympus Italia, Segrate, Italy), equipped with a Leica DFC 420 camera (Leica Microsystems, Milan, Italy). Electronic images were captured using a Leica Application Suite version 3.5.0 system (Leica Microsystems, Milan, Italy). For relative Oil Red O accumulation by spectrophotometry, after incubation with Oil Red O solution, stained cells were washed 3 times with distilled water, and the dye was eluted by the addition of 1 mL isopropanol for 15 minutes, with gentle shaking. A total of 100 µL of the eluted dye was removed from each sample and were transferred to a clean 96-well plate for reading the absorbance at 540 nm.

### 4.3. Filipin Staining

Filipin staining was performed as previously described [55]. Filipin staining was performed using Filipin complex (Sigma-Aldrich, catalog #F9765). Filipin stock solution (10mg/mL in PBS) was freshly prepared before the use. Cells were fixed in paraformaldehyde (4% solution) for 10 minutes and rinsed 3 times with PBS. Cells were stained with 1mL Filipin working solution (0.05 mg/mL in PBS) for 2 hours at room temperature, in the dark. Next, wells were rinsed 3 times with PBS to remove excess dye. Finally, cells were viewed in PBS through a fluorescence microscope using a UV filter set (340–380 nm excitation). Images were acquired at 20× magnification using an Axio Imager Z2 (Carl Zeiss, Jena, Germany) equipped with a charge-coupled device (CCD) camera controlled by the ISIS software (MetaSystems, Milano, Italy). Filipin quantification was calculated as the mean fluorescence intensity per cell area by using ImageJ Software for Windows.

### 4.4. Lysate Preparation and Western Blot Analysis

HepG2 lysate was performed as already reported [56]. Forty-eight hours after treatment, cells were lysed in 80 µL lysis buffer (0.25 M Tris pH 6.8, 10% SDS, phosphatase and protease inhibitor cocktails) by sonication (duty cycle 20%, output 3). Samples were then centrifuged at 10,000g for 10 min to remove cell debris. Protein concentration was assessed by the method of Lowry. Subsequently, Laemmli buffer was added to HepG2 lysates, and samples were boiled for 3 min before loading to the sodium dodecyl sulfate polyacrylamide gel electrophoresis (SDS-PAGE) for subsequent western blot analysis.

Western blot experiments were performed by slightly modifying the previously described protocol [8]. Briefly, proteins (30 μg) from HepG2 lysates were resolved on SDS-PAGE at 40 mA (constant current) for 60 min. Subsequently, proteins were transferred onto nitrocellulose membrane by using Trans-Blot Turbo Transfer System (Bio-Rad Laboratories, Milan, Italy). The nitrocellulose membrane was incubated at room temperature with 5% fat-free milk in Tris-buffered saline (0.138 M NaCl, 0.027 M KCl, 0.025 M Tris-HCl, and 0.05% Tween-20, pH 6.8), and probed at 4 °C overnight with the following primary antibodies: phopsho-HMGCR (Merck Millipore, #09-356, dilution 1:1000), HMGCR (Abcam, ab242315, dilution 1:1000), phospho-ACC (Sigma-Aldrich, SAB4503851, dilution 1:500), ACC (Sigma-Aldrich, SAB4501396, dilution 1:500), SR-B1 (Abcam, Cambridge, UK, ab52629, dilution 1:2000), LDLr (Abcam, ab30532, dilution 1:1000), NPC1 (Novus Biologicals, NB400-148, dilution 1:1000), TMEM97 (Novus Biologicals, Centennial, CO, USA, NBP1-30436, dilution 1:1000), SREBP-2 (Abcam, ab30682, dilution 1:1000), SREBP-1 (Santa Cruz Biotechnology, sc-8984, dilution 1:1000), alpha-Tubulin (Sigma-Aldrich, T6199, dilution 1:10000), vinculin (Sigma-Aldrich, V9131, dilution 1:20.000). Subsequently, membranes were probed for 1 hour with horseradish peroxidase conjugated secondary IgG antibodies (Bio-Rad Laboratories, Milan, Italy). Protein-antibody immunocomplexes onto nitrocellulose were visualized by using clarity ECL Western blotting (Bio-Rad Laboratories, Milan, Italy, #1705061), and chemiluminescence acquisition was carried out through ChemiDoc MP system (Bio-Rad Laboratories). Western blotting images were analyzed by ImageJ (National Institutes of Health, Bethesda, MD, USA) software for Windows. All samples were normalized for protein loading with alpha-Tubulin (chosen as a housekeeping protein). Recorded values were derived from the ratio between arbitrary units obtained from the protein band and the respective housekeeping protein.

### 4.5. Immunofluorescence Staining 

Immunofluorescence of HepG2 cells was performed by following the previously described protocol [17]. Cells were fixed in paraformaldehyde (4% in PBS) and incubated overnight with appropriate antibodies: HMGCR (Abcam, ab242315, dilution 1:100), SREBP-2 (Abcam, ab30682, dilution 1:100), SREBP-1 (Santa Cruz Biotechnology, Dallas, TX, USA, sc-8984, dilution 1:100), SR-B1 (Abcam, ab52629, dilution 1:100), LDLr (Santa Cruz Biotechnology, sc-11824, dilution 1:200), anti-Perilipin-2 (anti-Plin2) antibody (R&D Systems, #MAB76341, dilution 1:100). After incubation with primary antibodies, fixed cells were probed for 1 hour at room temperature with donkey anti-goat secondary antibody Alexa Fluor 488 (ThermoFisher Scientific, Milan, Italy, A-11055), goat anti-rabbit secondary antibody Alexa Fluor 555 (ThermoFisher Scientific, A27039) and goat anti-rabbit secondary antibody Alexa Fluor 488 (ThermoFisher Scientific, A-11008). Coverslips were mounted with Vectashield Antifade mounting medium with DAPI (Vector, H-1200) to visualize nuclear staining. The samples were examined at confocal microscopy (TCS SP8; Leica, Wetzlar, Germany). Images were captured using Leica TCS SP8 equipped with a 40 × 1.40–0.60 NA HCX Plan Apo oil BL objective at RT and Leica LAS X Software.

### 4.6. Statistical Analysis

All the results are expressed as mean ± standard deviation (SD). Normal distribution of the data was assessed by applying the Shapiro–Wilk test. Unpaired Student’s t test was performed to compare means between two experimental groups. When comparing three or more experimental groups, one-way analysis of variance (ANOVA) was carried out, followed by Tukey’s or Dunnett’s post hoc. *p* < 0.05 was considered to indicate a statistically significant difference. Statistical analysis and graph editing were performed using GRAPHPAD INSTAT3 (GraphPad, La Jolla, CA, USA) for Windows.

## Abbreviations

ACC	Acyl Coenzyme A carboxylase
BET	Bromodomain and extra-terminal domain
HDAC	histone deacetylases
HDL	high density lipoprotein
HMGCR	3-hydroxy-3-methylglutaryl Coenzyme A reductase
LDL	low density lipoprotein
LDLr	low density lipoprotein receptor
NPC1	Niemann-Pick type C1
ROS	reactive oxygen species
SR-B1	scavenger receptor class B type 1
SREBPs	sterol regulatory element binding proteins
TMEM97	transmembrane protein 97

## References

[1] Wakil S.J., Abu-Elheiga L.A. (2009). Fatty acid metabolism: target for metabolic syndrome. J. Lipid Res..

[2] Papackova Z., Cahová M. (2015). Fatty Acid Signaling: The New Function of Intracellular Lipases. Int. J. Mol. Sci..

[3] Trapani L., Segatto M., Pallottini V. (2012). Regulation and deregulation of cholesterol homeostasis: The liver as a metabolic “power station”. World J. Hepatol..

[4] Chen H.W. (1984). Role of cholesterol metabolism in cell growth. Fed. Proc..

[5] Yao C.H., Fowle-Grider R., Mahieu N.G., Liu G.Y., Chen Y.J., Wang R., Singh M., Potter G.S., Gross R.W., Schaefer J. (2016). Exogenous Fatty Acids Are the Preferred Source of Membrane Lipids in Proliferating Fibroblasts. Cell Chem. Biol..

[6] Nohturfft A., Zhang S.C. (2009). Coordination of Lipid Metabolism in Membrane Biogenesis. Annu. Rev. Cell Dev. Boil..

[7] Donaldson W.E. (1979). Regulation of fatty acid synthesis. Fed. Proc..

[8] Segatto M., Manduca A., Lecis C., Rosso P., Jozwiak A., Swiezewska E., Moreno S., Trezza V., Pallottini V. (2014). Simvastatin treatment highlights a new role for the isoprenoid/cholesterol biosynthetic pathway in the modulation of emotional reactivity and cognitive performance in rats. Neuropsychopharmacology.

[9] Segatto M., Trapani L., Lecis C., Pallottini V. (2012). Regulation of cholesterol biosynthetic pathway in different regions of the rat central nervous system. Acta Physiol..

[10] Eberlé D., Hegarty B., Bossard P., Ferré P., Foufelle F. (2004). SREBP transcription factors: master regulators of lipid homeostasis. Biochimie.

[11] Shen W.J., Azhar S., Kraemer F.B. (2018). SR-B1: A Unique Multifunctional Receptor for Cholesterol Influx and Efflux. Annu. Rev. Physiol..

[12] Cartocci V., Segatto M., Di Tunno I., Leone S., Pfrieger F.W., Pallottini V. (2016). Modulation of the Isoprenoid/Cholesterol Biosynthetic Pathway During Neuronal Differentiation In Vitro. J. Cell. Biochem..

[13] Cartocci V., Catallo M., Tempestilli M., Segatto M., Pfrieger F.W., Bronzuoli M.R., Scuderi C., Servadio M., Trezza V., Pallottini V. (2018). Altered Brain Cholesterol/Isoprenoid Metabolism in a Rat Model of Autism Spectrum Disorders. Neuroscience.

[14] Trapani L., Segatto M., Simeoni V., Balducci V., Dhawan A., Parmar V.S., Prasad A.K., Saso L., Incerpi S., Pallottini V. (2011). Short- and long-term regulation of 3-hydroxy 3-methylglutaryl coenzyme A reductase by a 4-methylcoumarin. Biochimie.

[15] Schulze H., Sandhoff K. (2011). Lysosomal Lipid Storage Diseases. Cold Spring Harb. Perspect. Boil..

[16] Wang C.-Y., Filippakopoulos P. (2015). Beating the odds: BETs in disease. Trends Biochem. Sci..

[17] Segatto M., Fittipaldi R., Pin F., Sartori R., Ko K.D., Zare H., Fenizia C., Zanchettin G., Pierobon E.S., Hatakeyama S. (2017). Epigenetic targeting of bromodomain protein BRD4 counteracts cancer cachexia and prolongs survival. Nat. Commun..

[18] Andrieu G.P., Shafran J.S., Deeney J.T., Bharadwaj K.R., Rangarajan A., Denis G.V. (2018). BET proteins in abnormal metabolism, inflammation, and the breast cancer microenvironment. J. Leukoc. Boil..

[19] Stathis A., Bertoni F. (2018). BET Proteins as Targets for Anticancer Treatment. Cancer Discov..

[20] Klein K. (2018). Bromodomain protein inhibition: a novel therapeutic strategy in rheumatic diseases. RMD Open.

[21] Hussong M., Börno S.T., Kerick M., Wunderlich A., Franz A., Sültmann H., Timmermann B., Lehrach H., Hirsch-Kauffmann M., Schweiger M.R. (2014). The bromodomain protein BRD4 regulates the KEAP1/NRF2-dependent oxidative stress response. Cell Death Dis..

[22] Siebel A.L., Trinh S.K., Formosa M.F., Mundra P.A., Natoli A.K., Reddy-Luthmoodoo M., Huynh K., Khan A.A., Carey A.L., Van Hall G. (2016). Effects of the BET-inhibitor, RVX-208 on the HDL lipidome and glucose metabolism in individuals with prediabetes: A randomized controlled trial. Metabolism.

[23] Sakamaki J.I., Wilkinson S., Hahn M., Tasdemir N., O’Prey J., Clark W., Hedley A., Nixon C., Long J.S., New M. (2017). Bromodomain Protein BRD4 Is a Transcriptional Repressor of Autophagy and Lysosomal Function. Mol. Cell.

[24] Li F., Yang C., Zhang H.-B., Ma J., Jia J., Tang X., Zeng J., Chong T., Wang X., He D. (2019). BET inhibitor JQ1 suppresses cell proliferation via inducing autophagy and activating LKB1/AMPK in bladder cancer cells. Cancer Med..

[25] Gilham D., Wasiak S., Tsujikawa L.M., Halliday C., Norek K., Patel R.G., Kulikowski E., Johansson J., Sweeney M., Wong N.C. (2016). RVX-208, a BET-inhibitor for treating atherosclerotic cardiovascular disease, raises ApoA-I/HDL and represses pathways that contribute to cardiovascular disease. Atherosclerosis.

[26] Zhang P., Wang D., Zhao Y., Ren S., Gao K., Ye Z., Wang S., Pan C.W., Zhu Y., Yan Y. (2017). Intrinsic BET inhibitor resistance in SPOP-mutated prostate cancer is mediated by BET protein stabilization and AKT-mTORC1 activation. Nat. Med..

[27] Filippakopoulos P., Qi J., Picaud S., Shen Y., Smith W.B., Fedorov O., Morse E.M., Keates T., Hickman T.T., Felletar I. (2010). Selective inhibition of BET bromodomains. Nature.

[28] Leng E., Xiao Y., Mo Z., Li Y., Zhang Y., Deng X., Zhou M., Zhou C., He Z., He J. (2018). Synergistic effect of phytochemicals on cholesterol metabolism and lipid accumulation in HepG2 cells. BMC Complement. Altern. Med..

[29] Mi Y., Tan D., He Y., Zhou X., Zhou Q., Ji S. (2018). Melatonin Modulates lipid Metabolism in HepG2 Cells Cultured in High Concentrations of Oleic Acid: AMPK Pathway Activation may Play an Important Role. Cell Biophys..

[30] Nagarajan S.R., Paul-Heng M., Krycer J.R., Fazakerley D.J., Sharland A.F., Hoy A.J. (2019). Lipid and glucose metabolism in hepatocyte cell lines and primary mouse hepatocytes: a comprehensive resource for in vitro studies of hepatic metabolism. Am. J. Physiol. Metab..

[31] Mehlem A., Hagberg C.E., Muhl L., Eriksson U., Falkevall A. (2013). Imaging of neutral lipids by oil red O for analyzing the metabolic status in health and disease. Nat. Protoc..

[32] Goldstein J.L., Brown M.S. (1987). Regulation of low-density lipoprotein receptors: implications for pathogenesis and therapy of hypercholesterolemia and atherosclerosis. Circulation.

[33] Yang X.-P., Amar M.J., Vaisman B., Bocharov A.V., Vishnyakova T.G., Freeman L.A., Kurlander R.J., Patterson A.P., Becker L.C., Remaley A.T. (2013). Scavenger receptor-BI is a receptor for lipoprotein(a). J. Lipid Res..

[34] Wojtanik K.M., Liscum L. (2003). The Transport of Low Density Lipoprotein-derived Cholesterol to the Plasma Membrane Is Defective in NPC1 Cells. J. Boil. Chem..

[35] Peake K.B., Vance J.E. (2010). Defective cholesterol trafficking in Niemann-Pick C-deficient cells. FEBS Lett..

[36] Ebrahimi-Fakhari D., Wahlster L., Bartz F., Werenbeck-Ueding J., Praggastis M., Zhang J., Joggerst-Thomalla B., Theiss S., Grimm D., Ory D.S. (2016). Reduction of TMEM97 increases NPC1 protein levels and restores cholesterol trafficking in Niemann-pick type C1 disease cells. Hum. Mol. Genet..

[37] Pesiri V., Totta P., Segatto M., Bianchi F., Pallottini V., Marino M., Acconcia F. (2015). Estrogen receptor α L429 and A430 regulate 17β-estradiol-induced cell proliferation via CREB1. Cell. Signal..

[38] Stock C.J.W., Michaeloudes C., Leoni P., Durham A.L., Mumby S., Wells A.U., Chung K.F., Adcock I.M., Renzoni E.A., Lindahl G.E. (2019). Bromodomain and Extraterminal (BET) Protein Inhibition Restores Redox Balance and Inhibits Myofibroblast Activation. Biomed. Res. Int..

[39] Roberts T.C., Etxaniz U., Dall’Agnese A., Wu S.-Y., Chiang C.-M., Brennan P.E., Wood M.J.A., Puri P.L. (2017). BRD3 and BRD4 BET Bromodomain Proteins Differentially Regulate Skeletal Myogenesis. Sci. Rep..

[40] Lu H., Talbot S., Robertson K.A., Watterson S., Forster T., Roy U., Ghazal P. (2015). Rapid proteasomal elimination of 3-hydroxy-3-methylglutaryl-CoA reductase by interferon-γ in primary macrophages requires endogenous 25-hydroxycholesterol synthesis. Steroids.

[41] Segatto M., Tonini C., Pfrieger F.W., Trezza V., Pallottini V. (2019). Loss of Mevalonate/Cholesterol Homeostasis in the Brain: A Focus on Autism Spectrum Disorder and Rett Syndrome. Int. J. Mol. Sci..

[42] Pahan K. (2006). Lipid-lowering drugs. Cell. Mol. Life Sci..

[43] Pipalia N.H., Cosner C.C., Huang A., Chatterjee A., Bourbon P., Farley N., Helquist P., Wiest O., Maxfield F.R. (2011). Histone deacetylase inhibitor treatment dramatically reduces cholesterol accumulation in Niemann-Pick type C1 mutant human fibroblasts. Proc. Natl. Acad. Sci. USA.

[44] Meaney S. (2014). Epigenetic regulation of cholesterol homeostasis. Front. Genet..

[45] Gaur V., Connor T., Sanigorski A., Martin S.D., Bruce C.R., Henstridge D.C., Bond S.T., McEwen K.A., Kerr-Bayles L., Ashton T.D. (2016). Disruption of the Class IIa HDAC Corepressor Complex Increases Energy Expenditure and Lipid Oxidation. Cell Rep..

[46] Lin Z., Bishop K.S., Sutherland H., Marlow G., Murray P., Denny W.A., Ferguson L.R. (2016). A quinazoline-based HDAC inhibitor affects gene expression pathways involved in cholesterol biosynthesis and mevalonate in prostate cancer cells. Mol. BioSyst..

[47] Ferrari A., Fiorino E., Giudici M., Gilardi F., Galmozzi A., Mitro N., Cermenati G., Godio C., Caruso D., De Fabiani E. (2012). Linking epigenetics to lipid metabolism: Focus on histone deacetylases. Mol. Membr. Boil..

[48] Bartz F., Kern L., Erz D., Zhu M., Gilbert D., Meinhof T., Wirkner U., Erfle H., Muckenthaler M., Pepperkok R. (2009). Identification of Cholesterol-Regulating Genes by Targeted RNAi Screening. Cell Metab..

[49] Sasaki M., Terao Y., Ayaori M., Uto-Kondo H., Iizuka M., Yogo M., Hagisawa K., Takiguchi S., Yakushiji E., Nakaya K. (2014). Hepatic overexpression of idol increases circulating protein convertase subtilisin/kexin type 9 in mice and hamsters via dual mechanisms: sterol regulatory element-binding protein 2 and low-density lipoprotein receptor-dependent pathways. Arterioscler. Thromb. Vasc. Biol..

[50] Kim Y.-M., Shin H.-T., Seo Y.-H., Byun H.-O., Yoon S.-H., Lee I.-K., Hyun N.-H., Chung H.-Y., Yoon G. (2010). Sterol Regulatory Element-binding Protein (SREBP)-1-mediated Lipogenesis Is Involved in Cell Senescence*. J. Boil. Chem..

[51] Segatto M., Trapani L., Di Tunno I., Sticozzi C., Valacchi G., Hayek J., Pallottini V. (2014). Cholesterol Metabolism Is Altered in Rett Syndrome: A Study on Plasma and Primary Cultured Fibroblasts Derived from Patients. PLoS ONE.

[52] Wen Y.-A., Xiong X., Zaytseva Y.Y., Napier D.L., Vallee E., Li A.T., Wang C., Weiss H.L., Evers B.M., Gao T. (2018). Downregulation of SREBP inhibits tumor growth and initiation by altering cellular metabolism in colon cancer. Cell Death Dis..

[53] Shi J., Vakoc C.R., Junwei S. (2014). The mechanisms behind the therapeutic activity of BET bromodomain inhibition. Mol. Cell.

[54] Gobbi G., Donati B., Valle I.F.D., Reggiani F., Torricelli F., Remondini D., Castellani G., Ambrosetti D.C., Ciarrocchi A., Sancisi V. (2019). The Hippo pathway modulates resistance to BET proteins inhibitors in lung cancer cells. Oncogene.

[55] Göritz C., Thiebaut R., Tessier L.-H., Nieweg K., Moehle C., Buard I., Dupont J.-L., Schurgers L.J., Schmitz G., Pfrieger F.W. (2007). Glia-induced neuronal differentiation by transcriptional regulation. Glia.

[56] Rocha J., Trapani L., Segatto M., Rosa P., Nogueira C., Zeni G., Pallottini V. (2013). Molecular Effects of Diphenyl Diselenide on Cholesterol and Glucose Cell Metabolism. Curr. Med. Chem..

